# Mechanism and therapeutic prospects of ferroptosis regulation through m6A in cancer

**DOI:** 10.3389/fcell.2026.1864925

**Published:** 2026-09-15

**Authors:** Yushuo Duan, Ziyi Xu, Jiahao Liang, Ruile Shen, Penghui Li

**Affiliations:** 1 Department of Neurology, The First Affiliated Hospital, and College of Clinical Medicine of Henan University of Science and Technology, Luoyang, China; 2 Henan Key Laboratory of Cancer Epigenetics, Cancer Institute, The First Affiliated Hospital, and College of Clinical Medicine of Henan University of Science and Technology, Luoyang, China; 3 Department of Gastrointestinal Surgery, The First Affiliated Hospital, and College of Clinical Medicine of Henan University of Science and Technology, Luoyang, China

**Keywords:** ferroptosis, m6A methylation, therapeutic target, therapy resistance, tumor immune microenvironment, tumor progression

## Abstract

N6-methyladenosine (m6A), as the most abundant epitranscriptomic modification in eukaryotes, profoundly influences the metabolic fate of RNA. Meanwhile, ferroptosis, a regulated cell death modality driven by iron-dependent lipid peroxidation, has become a key component in tumor metabolic reprogramming. Increasing evidence suggests a deep interaction between m6A modification and ferroptosis, which plays a critical role in tumor occurrence and development, and treatment response. This review systematically elucidates the multidimensional regulatory mechanisms of m6A on ferroptosis regulatory factors, covering post-transcriptional modifications of the System Xc^−^/GSH/GPX4 antioxidant axis, iron metabolism-related proteins, and key enzymes of lipid peroxidation. It analyzes the dual role of the m6A-ferroptosis axis in tumor suppression and promotion, and then explores the function of this regulatory network in reshaping the tumor immune microenvironment and mediating treatment resistance. Finally, it looks forward to the translational potential of the m6A-ferroptosis interactive network as a novel biomarker and combination therapy target. Future research should integrate multi-omics and cutting-edge technologies to deeply analyze the spatiotemporal dynamic mechanism of this regulatory network in the occurrence and development of tumors, providing theoretical basis and new research directions for precise intervention in this interdisciplinary field.

## 1 Introduction

The progression of malignant tumors and treatment resistance has long been a core challenge in successful cancer treatment worldwide (Sun et al., 2012; Lytle et al., 2018). Tumor heterogeneity leads to drug resistance in conventional therapies, which is especially prominent in solid tumors with inactivated apoptotic pathways (Akumaga et al., 2025). In this context, exploring non-apoptotic cell death pathways has emerged as a new direction to overcome resistance (Shang et al., 2026). Ferroptosis, as a novel regulatory cell death mechanism driven by iron dependence and lipid peroxidation, has become a research hotspot in the field of tumor therapy due to its unique metabolic characteristics and immunogenicity (Kang et al., 2023; Kang et al., 2026). The essence of this process is a lipid peroxidation cascade reaction triggered by the imbalance of intracellular redox homeostasis, with three key regulatory aspects: iron metabolism disorder, lipid peroxidation accumulation, and antioxidant system dysfunction (Alves et al., 2025; Mou et al., 2019). Therefore, ferroptosis as a typical metabolism-related cell death mechanism, with its deep metabolic and stress interaction characteristics, opens up multi-level therapeutic windows for the precise targeting of tumor metabolic vulnerabilities.

N6-methyladenosine (m6A) is the most abundant and dynamically reversible post-transcriptional modification in eukaryotic messenger RNA (mRNA) (Jiang et al., 2021). This modification is regulated by three classes of functional proteins: “Writers” are responsible for catalyzing the methylation modification; “Erasers” execute demethylation; and “Readers” specifically recognize m6A sites, thereby regulating RNA splicing, nuclear export, stability, translation, and degradation, and are widely involved in biological processes such as cell cycle, differentiation, metabolism, and stress response (Ahlawat et al., 2026; Wen et al., 2026). In tumorigenesis and development, abnormal m6A modification can drive tumor progression and promote treatment resistance through various mechanisms, including enhancing oncogene expression, inhibiting tumor suppressor gene function, mediating metabolic reprogramming, and reshaping the tumor immune microenvironment (TIME), thus becoming an important therapeutic target with significant translational potential in epitranscriptomics (Qu et al., 2020; Tan et al., 2021; Zheng et al., 2022; Wang D. et al., 2023).

An increasing body of evidence suggests that the m6A modification intersects with ferroptosis in core regulatory pathways: m6A, as a key node in epitranscriptional regulation, can precisely coordinate the expression of genes related to iron metabolism, antioxidant defense and lipid metabolism, integrating the metabolic reprogramming of tumor cells with oxidative stress signals, thereby dynamically regulating sensitivity to ferroptosis. Although this m6A-ferroptosis axis plays critical roles in tumor biology, most prior reviews only discuss single tumor subtypes or partial signaling axes (Wang L. et al., 2025).

Based on this foundation, this review systematically elucidates the multi-dimensional mechanisms of m6A-regulated ferroptosis across diverse common malignancies and integrates its key roles in tumor progression, immune microenvironment remodeling, and drug resistance. By delineating core regulatory axes and emerging targeted intervention strategies alongside prospective translational hurdles, this review provides a comprehensive framework for precision cancer therapies based on the m6A-ferroptosis axis.

## 2 N^6^-methyladenosine (m6A)

M6A is the most abundant and conserved epitranscriptomic modification in eukaryotic mRNA, specifically referring to the methylation of the nitrogen atom at the sixth position of adenine (Perry et al., 1975; Zhu et al., 2020). This modification specifically recognizes the RRACH motif and is highly enriched in stop codons, 3′untranslated regions (3′UTR), and long internal exon regions (Dominissini et al., 2012; Zhang et al., 2017). In fact, the m6A modification not only regulates mRNA but is also widely present in various non-coding RNAs (ncRNAs) such as microRNA (miRNA), long non-coding RNA (lncRNA) and circular RNA (circRNA) (Alarcón et al., 2015; Dai et al., 2018; Lin et al., 2022). M6A is widely involved in the entire RNA metabolism process, including processing, transport, translation, and degradation, and is a key player in regulating cellular life activities and physiological functions (Ahlawat et al., 2026). This dynamic and reversible modification is orchestrated by the coordinated actions of “Writers,” “Erasers,” and “Readers,” whose interplay constitutes a multilayered regulatory network schematically depicted in Figure 1.

**FIGURE 1:**
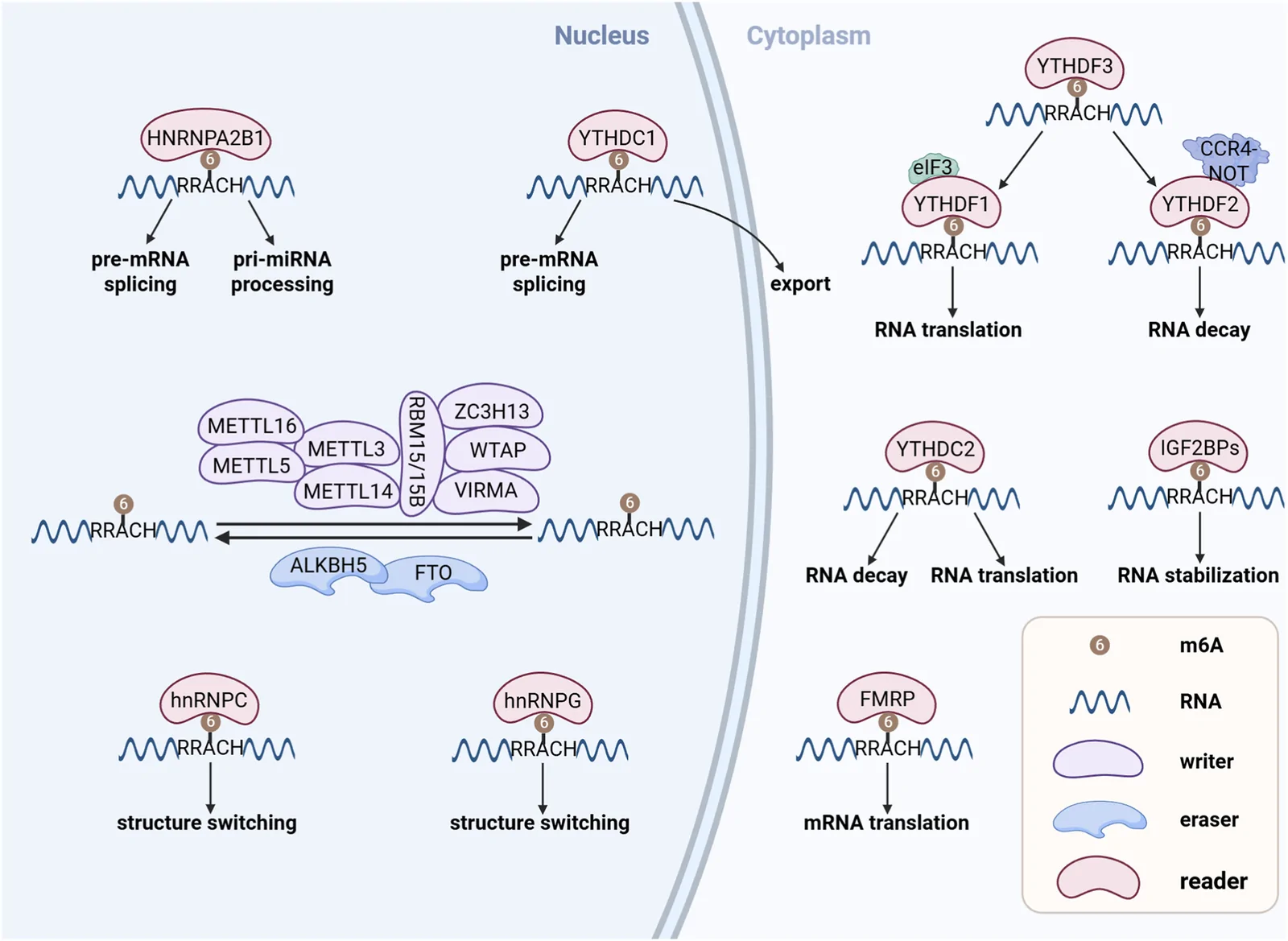
Function of m6A modification. The m6A modification is a dynamic and reversible process. The establishment of this modification relies on a methyltransferase complex centered around METTL3-METTL14, aided by factors such as WTAP, VIRMA, ZC3H13, and RBM15/15B for co-catalysis, while its erasure is mediated by demethylases FTO and ALKBH5. At the functional execution level, the m6A modification is specifically recognized by “Readers,” including YTHDF1-3, YTHDC1-2, IGF2BP1-3, and certain hnRNP family proteins (such as hnRNP C, hnRNP G, HNRNPA2B1). These “Readers” determine the fate of target RNA in processes such as splicing, nuclear export, stability, translation, or degradation by binding to m6A sites, thereby achieving the precise regulation of gene expression.

### 2.1 Writers

The m6A modification is primarily mediated by the methyltransferase complex (MTC), which is catalyzed by the heterodimer of methyltransferase-like 3 (METTL3) and METTL14 as the core catalytic unit (He et al., 2019). METTL3 is responsible for catalyzing adenosine methylation, while METTL14 recognizes the RRACH motif and maintains the structural stability of the complex (Wang et al., 2017; Zeng et al., 2020). To ensure the spatiotemporal specificity of the modification, various regulatory subunits are involved: the Wilms tumor 1-associated protein (WTAP) and the Vir-like m6A methyltransferase-associated (VIRMA) guide the complex to specific regions such as nuclear speckles and 3′UTR (Ping et al., 2014; Yue et al., 2018); the zinc finger CCCH domain-containing protein 13 (ZC3H13) enhances its efficacy by regulating the nuclear localization of the complex (Wen et al., 2018); the RNA-binding motif protein 15 (RBM15) and its paralog RBM15B assist in recruiting the complex to specific sequences (Wang et al., 2020; Patil et al., 2016). Besides, enzymes such as METTL16 and METTL5 operate independently of this complex, which are specifically responsible for the m6A modification of certain RNA molecules like U6 small nuclear RNA (snRNA) and ribosomal RNA (rRNA) (Pendleton et al., 2017; Peng et al., 2022).

### 2.2 Erasers

In contrast to “Writers”, demethylases fat mass and obesity-associated protein (FTO) and AlkB homolog 5 (ALKBH5) can remove methyl groups, imparting a dynamic reversible characteristic to m6A modification (Wei et al., 2022; Qu et al., 2022).

### 2.3 Readers

The biological functions of m6A ultimately depend on the specific recognition by “Readers” and the recruitment of downstream effectors. Members of the YTH domain family (including YTHDF1/2/3 and YTHDC1/2) serve as the main effectors (Shi et al., 2021). Nuclear YTHDC1 regulates mRNA splicing and export (Chen and Wong, 2020), while cytoplasmic YTHDC2 enhances target mRNA translation and reduces its abundance (Hsu et al., 2017). YTHDF2 promotes mRNA degradation by recruiting the CCR4-NOT complex (Du et al., 2016; Lee et al., 2020), and YTHDF1 interacts with eukaryotic initiation factor 3 (eIF3) to enhance translation efficiency (Wang et al., 2015). Conversely, insulin-like growth factor 2 mRNA-binding proteins (including IGF2BP1/2/3) enhance mRNA stability by binding to m6A sites, thereby promoting gene expression (Chen and Wong, 2020). Furthermore, the m6A modification can indirectly regulate the binding of heterogeneous nuclear ribonucleoproteins (such as hnRNP C, hnRNP G, and HNRNPA2B1) by altering the RNA secondary structure and exposing specific binding sites, thereby affecting the processing and splicing of precursor mRNA (pre-mRNA) (Liu et al., 2017; Xu P. et al., 2023). HNRNPA2B1 can also promote the processing and maturation of primary miRNA (pri-miRNA) by recognizing m6A modification (Li R. et al., 2024). In the nervous system, Fragile X Messenger Ribonucleoprotein 1 (FMRP) also acts as a reading protein involved in the fine regulation of neuronal mRNA translation (Darnell and Richter, 2012).

In recent years, the m6A modification was found to play a key role in dynamically regulating RNA metabolism and function, particularly in stem cell differentiation and embryo development, and especially in oncogenesis and drug resistance (Zhang et al., 2021; Deng et al., 2023). While these findings reveal the multidimensional complexity of epitranscriptomic regulation, the phenotypic outcomes of m6A dysregulation in cancer rely heavily on its coordinated control over downstream metabolic vulnerabilities and cell death programs. In particular, emerging evidence underscores ferroptosis as a crucial metabolic process intimately governed by m6A-mediated epitranscriptomic reprogramming. Therefore, systematically deciphering the fundamental regulatory machinery of ferroptosis is essential to understanding how the m6A-ferroptosis interplay dictates tumor progression and therapeutic susceptibility.

## 3 Regulatory mechanisms of ferroptosis

Ferroptosis, a novel form of programmed cell death (PCD) that was first named by Dixon et al., in 2012, is distinct from apoptosis, necrosis, autophagy, and pyroptosis (Mou et al., 2019; Dixon et al., 2012; Miao et al., 2023). Its core biochemical feature lies in the iron-dependent generation of reactive oxygen species (ROS) and the lethal accumulation of lipid peroxides (Bell et al., 2024). The onset of ferroptosis, driven by the collapse of intracellular redox homeostasis and tightly regulated by the interplay of iron metabolism, lipid peroxidation, and antioxidant system dysfunction, is comprehensively illustrated in Figure 2.

**FIGURE 2:**
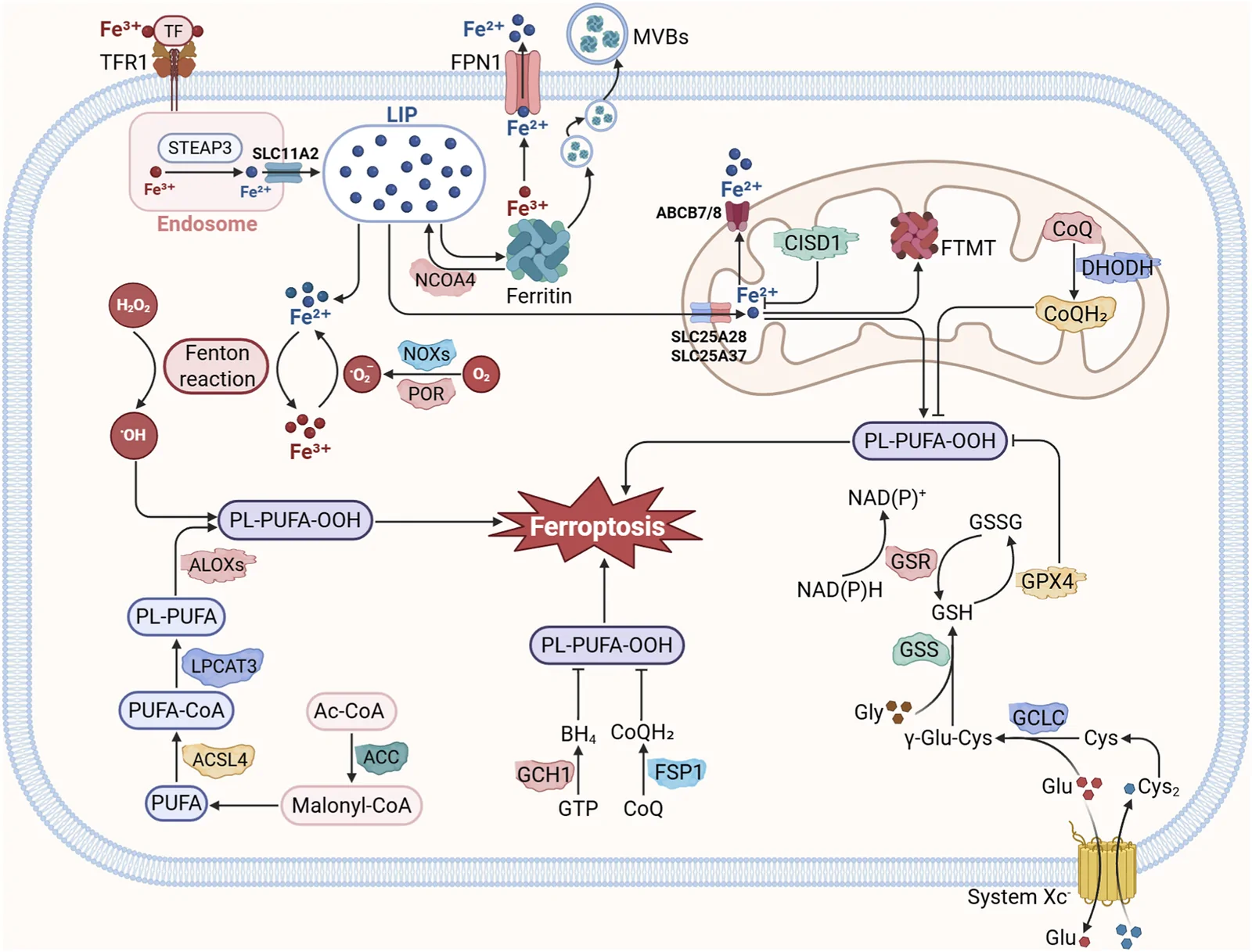
Regulatory mechanisms of ferroptosis. Ferroptosis is a form of iron-dependent, lipid peroxidation-driven cell death, which is determined by four core processes: In terms of iron metabolism, Tf endocytoses iron ions through its receptor TfR1, which are transported to the cytoplasm by SLC11A11 after reduction, leading to iron overload and triggering the Fenton reaction, producing ROS free radicals. The PUFA mediated by ACSL4/LPCAT3 undergo irreversible lipid peroxidation under attack by ROS, damaging membrane integrity. The antioxidant system centered on the System Xc^−^/GSH/GPX4 axis (including compensatory pathways such as FSP1) loses function, causing uncontrolled peroxidative damage. Mitochondria as the regulatory hub maintain iron homeostasis by importing iron through SLC25A37/28, storing it via FTMT or exporting it through ABCB7/8, and rely on CISD1 to regulate redox balance, with dysfunction directly triggering ferroptosis. These processes are interconnected, forming a complete regulatory network.

### 3.1 Iron metabolism

Iron is an essential trace element for life activities, and the imbalance of its metabolic homeostasis is a key driver of ferroptosis (Wu et al., 2025). Fe^3+^ in the blood binds to transferrin (TF), which is recognized by transferrin receptor 1 (TFR1) and endocytosed into endosomes, where it is reduced to Fe^2+^ by six-transmembrane epithelial antigen of the prostate 3 (STEAP3) and transported to the cytoplasm by solute carrier family 11 member 2 (SLC11A2), forming a labile iron pool (LIP) (Shi et al., 2024). When LIP is overloaded, Fe^2+^ catalyzes the production of hydroxyl radicals from hydrogen peroxide (H_2_O_2_) through the Fenton reaction, directly initiating lipid peroxidation, which is the core mechanism triggering ferroptosis (Zhu et al., 2024; Zhu et al., 2022). To balance iron utilization and toxicity, cells have established a tight regulatory system. Under normal circumstances, excess iron is stored in ferritins (Li S. et al., 2024). However, under ferroptosis signaling, ferritinophagy mediated by nuclear receptor coactivator 4 (NCOA4) degrades ferritins, releasing free iron to exacerbate the cell death process (Li et al., 2021; Qin et al., 2021). Conversely, cells can export iron through ferroportin 1 (FPN1) (Shpyleva et al., 2011) or exocytose ferritins via multivesicular bodies (MVBs) (Brown et al., 2019; Truman-Rosentsvit et al., 2018), thereby reducing intracellular iron toxicity.

### 3.2 Lipid peroxidation

The core hallmark of ferroptosis is lipid peroxidation, which begins with the accumulation of phospholipid-polyunsaturated fatty acids (PL-PUFAs) in the cell membrane (Zhou et al., 2024). The sensitivity of cells to ferroptosis is primarily determined by the content of PL-PUFAs, a process that relies on the cooperation of acyl-CoA synthetase long-chain family member 4 (ACSL4) and lysophosphatidylcholine acyltransferase 3 (LPCAT3). The former activates free PUFAs, while the latter incorporates them into membrane phospholipids (Zhou et al., 2024; Li Y. et al., 2025). In addition, acetyl-CoA carboxylase (ACC) is crucial for this process by catalyzing the production of the PUFA synthesis precursor malonyl-CoA (Lei et al., 2022). Subsequently, lipid peroxidation is initiated through certain key enzyme-catalyzed pathways. Specifically, arachidonic acid lipoxygenases (ALOXs) directly oxidize membrane PL-PUFAs to form lipid peroxides (Kühn and Borchert, 2002); simultaneously, cytochrome P450 oxidoreductase (POR) and others indirectly promote peroxidation by generating ROS (Zou et al., 2020). The nicotinamide adenine dinucleotide phosphate (NADPH) oxidases (NOXs) system enhances the overall oxidative stress level by producing superoxide radicals, creating and amplifying the reactive environment for peroxidation (Gorrini et al., 2013; Lambeth and Neish, 2014). ALOXs, POR and NOXs together form a synergistic attack network that drives the explosion of lipid peroxidation. Notably, in tumor biology, cancer cells often downregulate the levels of peroxidized PL-PUFAs through metabolic reprogramming to evade ferroptosis, highlighting the central role of this regulatory network in tumor progression and treatment resistance.

### 3.3 Dysfunction of the antioxidant system

Tumor cells resist ferroptosis by constructing a multi-layered, synergistic antioxidant defense network, which is central to their malignant progression and treatment resistance (Gorrini et al., 2013). The core of this network is the classical system Xc^−^/glutathione (GSH)/glutathione peroxidase 4 (GPX4) pathway. System Xc- is composed of SLC7A11 and SLC3A2, responsible for the uptake of cystine (Huang et al., 2005; Koppula et al., 2018). Cystine is reduced to cysteine within cells. Cysteine then combines with glutamate in a reaction catalyzed by glutamate-cysteine ligase catalytic subunit (GCLC) to form γ-glutamylcysteine (γ-Glu-Cys). Subsequently, glutathione synthetase (GSS) incorporates glycine to synthesize GSH (Wu et al., 2025). GPX4 then utilizes GSH to reduce toxic lipid hydroperoxides into harmless lipid alcohols, thereby blocking the chain reaction of lipid peroxidation (Li S. et al., 2024; Zhou et al., 2024). This process consumes GSH and produces oxidized GSH (GSSG) that then relies on NADPH to be regenerated back to GSH through glutathione reductase (GSR) to maintain the antioxidant cycle (Weaver and Skouta, 2022). In addition to the aforementioned core pathway, there exists a non-GPX4 dependent defense system. Its most important member is the ferroptosis suppressor protein 1 (FSP1)-coenzyme Q10 (CoQ10)-NAD(P)H pathway, wherein FSP1, located at the plasma membrane, can reduce CoQ10 to ubiquinol-10 (CoQ10H2), which acts as a lipophilic antioxidant to directly scavenge lipid radicals (Doll et al., 2019; Bersuker et al., 2019). Furthermore, dihydroorotate dehydrogenase (DHODH) in the mitochondria and the GTP cyclohydrolase 1 (GCH1)-tetrahydrobiopterin (BH4) system in the cytoplasm also constitute an independent defense against lipid peroxidation (Xu L. et al., 2023; Gao M. et al., 2022). These pathways are precisely regulated, and once disrupted (such as by inhibition of System Xc^−^, inactivation of GPX4, or loss of compensatory pathways), it will lead to lipid peroxidation and trigger ferroptosis.

Mitochondria, as the hub of this network, regulate the integration and amplification of ferroptosis signals. Iron enters the mitochondrial matrix through translocator proteins such as SLC25A37 or SLC25A28 (Dixon and Olzmann, 2024; Kang et al., 2019). Mitochondrial ferritin (FTMT) is responsible for storing iron to reduce its reactivity and exports iron via ATP-binding cassette subfamily B member 7/8 (ABCB7/8) to maintain iron homeostasis (Napier et al., 2005; Nikpour et al., 2013; Zheng et al., 2025). The core function of CDGSH iron-sulfur domain-containing protein 1 (CISD1) is to regulate mitochondrial iron homeostasis and redox reactions. The loss of CISD1 function leads to mitochondrial iron accumulation and lipid peroxidation, thereby driving ferroptosis (Zhou et al., 2024; Lipper et al., 2019).

In recent years, ferroptosis has become an important direction in tumor-related research and treatment. Studies have shown that the ferroptosis pathway not only dominates cell fate but also deeply participates in tumor progression, treatment resistance, and the remodeling of the immune microenvironment (Friedmann et al., 2019). Therefore, selectively inducing ferroptosis in tumor cells provides a key clinical translation strategy to overcome multidrug resistance and break tumor immune tolerance.

## 4 M6A regulation of ferroptosis and its role in cancer

In recent years, an increasing number of studies have revealed a close molecular crosstalk between m6A modification and ferroptosis. The m6A modification not only directly drives the metabolic reprogramming of tumor cells by regulating the mRNA metabolism of ferroptosis-related genes but also dynamically reshapes the cellular infiltration and functional status of the TIME through the immunogenic characteristics of ferroptosis. This multidimensional regulatory network provides a new perspective for comprehensively analyzing the mechanisms of cancer occurrence and development, immune evasion, and treatment resistance.

### 4.1 Dual role in tumor development

Existing evidence highlights that, instead of a single linear pattern, the regulation of ferroptosis via m6A modification has significant context dependence and is based on tumor heterogeneity (Wen et al., 2026; Tao et al., 2024; Wei et al., 2024). This epigenetic modification plays a bidirectional regulatory role in tumor development, with specific effects depending on the specific expression of m6A regulatory factors and the functional properties of downstream target genes.

#### 4.1.1 Role in tumor progression

Upregulation of core antioxidant hubs (SLC7A11 and GPX4) represents a primary anti-ferroptotic strategy. In lung adenocarcinoma (LUAD), METTL3 recruits YTHDF1 to enhance the stability and translation of SLC7A11 (Xu Y. et al., 2022). In hepatoblastoma, METTL3 stabilizes SLC7A11 mRNA via IGF2BP1 to prevent deadenylation (Liu L. et al., 2022), or it indirectly activates SLC7A11 by upregulating tyrosine 3-monooxygenase/tryptophan 5-monooxygenase activation protein epsilon (YWHAE) through IGF2BP2 (Zhou et al., 2025). In breast cancer (BC), METTL3 promotes SLC7A11 mRNA splicing and translation by facilitating the interaction between FMRP and hnRNPM, thereby inhibiting ferroptosis and driving tumor progression (Wang et al., 2024a). The NF-kappaB activating protein (NKAP) recognizes the m6A modification on SLC7A11 mRNA mediated by METTL3 and recruits splicing factor proline and glutamine-rich (SFPQ) to promote its correct splicing and maturation, thereby maintaining SLC7A11 expression and inhibiting ferroptosis, ultimately promoting the progression of glioblastoma (Sun et al., 2022). Notably, in HPV-positive cervical cancer, the low expression of YTHDC2 promotes the translation of SLC7A11 mRNA by unwinding its 5′UTR, thereby inhibiting ferroptosis and promoting tumor proliferation (Ren L. et al., 2025). This complex multidimensional regulatory mechanism collectively blocks ferroptosis and promotes malignant tumor progression.

The m6A modification also constructs an anti-ferroptosis barrier by regulating GPX4. Among them, IGF2BP3 enhances glioma cell resistance to ferroptosis by binding to and stabilizing GPX4 mRNA, thereby promoting tumor growth (Deng et al., 2024). METTL14 inhibits ferroptosis in non-small cell lung cancer (NSCLC) by modifying GPX4 mRNA and enhancing its stability, improving the cell’s ability to clear lipid peroxides (Lou et al., 2024). Protein kinase A (PKA) induces ALKBH5 degradation through phosphorylation, enhancing GPX4 mRNA stability and forming a broad-spectrum anti-ferroptosis mechanism in fibrosarcoma, melanoma and NSCLC (Zhao et al., 2026). Cross-cancer comparative analysis reveals that the same writer METTL3 engages distinct readers (YTHDF1, IGF2BP1/2, and FMRP-hnRNPM) to regulate common effectors (SLC7A11 and GPX4) across malignancies. This pattern underscores the tissue-specific plasticity of m6A-ferroptosis regulation, offering versatile entry points for precision therapy.

In addition to regulating ferroptosis-related genes, the m6A modification also drives tumor progression by regulating the metabolism and function of ncRNA. At the lncRNA level, high-density lipoprotein binding protein (HDLBP) cooperates with YTHDF2 to stabilize lncFAL, which competitively inhibits the ubiquitination degradation of FSP1, enhancing its stability to suppress ferroptosis in hepatic carcinoma cells (Yuan et al., 2022). In colorectal cancer (CRC), METTL3 activates the E3 ligase tripartite motif containing 21 (TRIM21)/IGF2BP2/forkhead box M1 (FOXM1) positive feedback loop by stabilizing lncRNA ABHD11-AS1 (Bian et al., 2024), while HNRNPC activates the nuclear factor erythroid 2-related factor 2 (Nrf2)/GPX4 pathway by stabilizing lncRNA AC145207.5 (Liu et al., 2025a). In cervical neoplasms, the METTL3-mediated high expression of lncRNA COTE-1 inhibits autophagy-dependent ferroptosis (Min et al., 2025). Furthermore, ALKBH5 promotes the progression of LUAD by downregulating lncRNA RBMS3-AS3, thereby relieving the inhibition on the heterogeneous nuclear ribonucleoprotein D-like (HNRNPDL)/zinc finger E-box binding homeobox 1 (ZEB1)/GPX4 (Ge W. et al., 2025). At the circRNA level, circEZH2 is upregulated through the IGF2BP2-mediated m6A positive feedback loop and drives lipid metabolism reprogramming and ferroptosis resistance in carcinoma of the gallbladder via the miR-556-5p/stearoyl-CoA desaturase (SCD1) axis, thus promoting tumor progression (Tong et al., 2024). In bladder cancer, METTL3-mediated m6A modification promotes the high expression of circCD2AP, which activates the forkhead box C1 (FOXC1)/Parkinson’s disease protein 7 (PARK7) pathway through YTHDC1 transport, thereby inhibiting ferroptosis and driving tumor progression (Wang et al., 2025b). Notably, the same m6A writers target distinct ncRNAs across cancer types, such as METTL3 via ABHD11-AS1 in colorectal cancer and COTE-1 in cervical cancer, yet converge on ferroptosis execution, revealing functional convergence with molecular diversity. These findings establish the m6A-ncRNA axis as a critical ferroptosis evasion mechanism, suggesting that targeting this axis may restore ferroptosis sensitivity.

In terms of iron metabolism regulation, the m6A modification builds a key ferroptosis defense mechanism by limiting the accumulation of free iron within cells. In nasopharyngeal carcinoma (NPC) (Yuan et al., 2025) and lung cancer (Diao et al., 2023), IGF2BP2 and YTHDF1 effectively reduce intracellular iron levels by stabilizing ceruloplasmin (CP) mRNA and promoting the translation of ferritin heavy chain (FTH). In hepatocellular carcinoma (HCC), METTL16 stabilizes SUMO-specific protease 3 (SENP3) mRNA through IGF2BP2 to maintain the expression of lactotransferrin (LTF) (Wang J. et al., 2024). In terms of mitochondrial function regulation, METTL16 promotes tumor growth by stabilizing activating transcription factor 4 (ATF4) mRNA, inhibiting ferroptosis in cholangiocarcinoma cells and maintaining mitochondrial function (Zhao et al., 2025).

Finally, this pro-tumorigenic network is dynamically triggered by diverse upstream cues. The environmental carcinogen Benzo [a]pyrene (BaP) upregulates YTHDF1 to enhance SLC7A11 and FTH1 translation, thereby inhibiting ferroptosis and driving the malignant transformation and invasion of bronchial epithelial cells (Wang et al., 2024c). The inflammatory factor interleukin-6 (IL-6) promotes the expression of KIAA1429 by activating the Janus kinase 1 (JAK1)/signal transducer and activator of transcription 3 (STAT3) pathway, thereby accelerating the degradation of DNA damage-inducible transcript 3 (DDIT3) mRNA, ultimately inhibiting ferroptosis and driving the progression of endometrial cancer (EC) (Shen X. et al., 2025). In addition, protein and histone modifications also play a core inductive role. The acetylation of Jumonji domain-containing protein 6 (JMJD6) and the H3K36me3 modification mediated by SET domain-containing 5 (SETD5) can activate METTL14 to stabilize SLC3A2 mRNA or enhance GPX4 expression in NSCLC (Chen et al., 2025; Liu X. et al., 2025). These upstream mechanisms reshape m6A modifications, comprehensively activating the anti-ferroptosis network, enabling tumor cells to resist oxidative stress and significantly driving their proliferation, invasion and metastasis.

#### 4.1.2 Role in tumor suppression

Regarding the role of m6A in tumor suppression, on the one hand, by inhibiting the expression of anti-ferroptosis genes, m6A disrupts the antioxidant defense system of tumor cells and induces ferroptosis. In gastric cancer (GC), methionine restriction (MR) can downregulate METTL3 expression, reducing its protective effect on Fanconi anemia complementation group D2 (FANCD2) mRNA stability, leading to the collapse of the antioxidant system and inducing ferroptosis (Xin et al., 2025). In endometrial carcinoma, lncRNA NORAD promotes the downregulation of GPX4 and induces ferroptosis by binding to and inhibiting FTO, thereby relieving the demethylation protective effect of the latter on GPX4 mRNA, ultimately suppressing tumor growth (Ke et al., 2025). Similarly, in CRC, v-AKT murine thymoma viral oncogene homolog (AKT) signaling inhibition can downregulate FTO expression, thereby increasing the m6A modification level of GPX4 mRNA, making it recognized and degraded by YTHDF2, thus reversing the tumor’s resistance to ferroptosis (Zhang G. et al., 2023).

On the other hand, the m6A modification and its regulatory network directly drive tumor cell death by upregulating factors related to ferroptosis. In GC, METTL14 activates the ferroptosis program by stabilizing sirtuin 5 (SIRT5) mRNA, thereby inhibiting tumor cell proliferation, migration and invasion (Zhang et al., 2025a). Additionally, ncRNAs also play an important role in upstream regulation: in glioma, lncRNA GDNF-AS1 reduces the overall m6A modification level by inhibiting METTL3 transcription, thereby relieving the suppression of NCOA4, promoting ferritinophagy, and ultimately inducing ferroptosis (Zhou et al., 2026). Furthermore, in BC brain metastasis, circSCYL2 induces ferroptosis and inhibits tumor metastasis by competitively binding to hnRNPA2B1, blocking its mediated H3K9me3 modification to relieve the transcriptional repression of ACSL4 (Yuan et al., 2026).

In summary, given the bidirectional role of the m6A modification in regulating ferroptosis and tumor progression, future treatment strategies should not generally inhibit or activate the m6A modification but deeply analyze its precise mechanism of action in specific tumor types and develop selective modulators to achieve more precise cancer treatment. As shown in Figure 3, m6A modification regulates ferroptosis and tumor fate through dual pathways: in the pro-tumorigenic context, m6A establishes an anti-ferroptosis barrier by upregulating SLC7A11/GPX4, and modulates tumor metabolic adaptability by regulating non-coding RNAs (such as lncFAL, circCD2AP) and iron metabolism dynamics (such as FTH), driving tumor progression; in the anti-tumorigenic context, m6A directly induces tumor cell death by inhibiting anti-ferroptosis genes or upregulating pro-ferroptosis factors (such as ACSL4), thereby disrupting the antioxidant defense system. The net outcome of this bidirectional regulation is highly dependent on the tumor type and microenvironment. Additionally, the cross-regulatory mechanisms between m6A and other cell death pathways (such as apoptosis, pyroptosis, and autophagy) merit further investigation.

**FIGURE 3:**
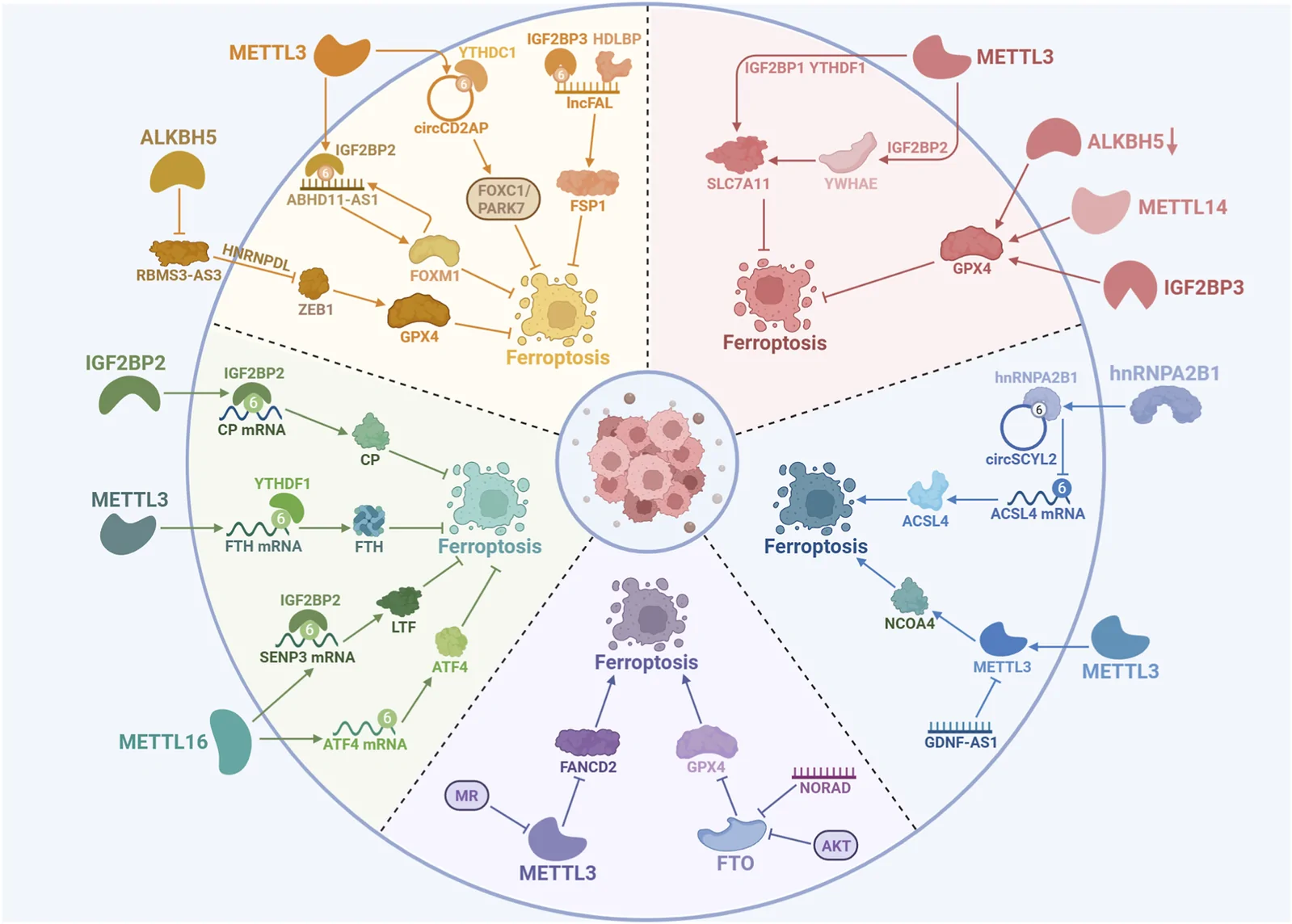
The m6A modification regulates the dual role of ferroptosis in tumor development. M6A drives tumor progression by inhibiting ferroptosis: METTL3/METTL14, IGF2BP1/2/3 and ALKBH5 work synergistically to directly enhance the mRNA stability and translation efficiency of SLC7A11 and GPX4. M6A promotes tumor progression by regulating the metabolism and function of ncRNAs (such as lncFAL, circCD2AP). By regulating iron metabolism-related proteins (such as CP, FTH) and mitochondrial stress response (ATF4), m6A limits the accumulation of free iron and oxidative stress in cells, thereby inhibiting ferroptosis. M6A inhibits tumor growth by inducing ferroptosis: Low METTL3 expression or the inhibition of FTO activity can downregulate FANCD2 and GPX4, weakening the antioxidant defense of tumor cells and sensitizing them to ferroptosis. Specific ncRNAs (lncRNA GDNF-AS1, circSCYL2) interfere with the m6A regulatory network, relieving the inhibition on NCOA4 and ACSL4, thereby activating the ferroptosis program and inhibiting tumor growth.

### 4.2 Impact on the tumor immune microenvironment

In response to the limitations of therapy with immune checkpoint inhibitors (ICIs), inducing ferroptosis has become an important direction for reshaping the TIME (Demaria et al., 2016; Ding et al., 2023; Demuynck et al., 2021). Recent studies have shown that m6A plays a vital role of “molecular switch” in the initiation of ferroptosis. This regulation not only directly drives tumor cells to undergo lipid peroxidation death but also inhibits CD8^+^ T cell function by releasing specific lipid metabolites and immune signaling molecules, and promotes the polarization of M2 macrophages and the differentiation of regulatory T (Treg) cells, thereby driving immune evasion.

#### 4.2.1 CD8^+^ T cells

CD8^+^ T cells, as the core cytotoxic lymphocytes of the adaptive immune system, can specifically recognize and directly kill tumor cells, serving as the main effector cells in immunotherapy (McKenzie et al., 2022; Farhood et al., 2019). The m6A modification network profoundly influences the activation and anti-tumor functions of CD8^+^ T cells via regulating the sensitivity of tumor cells to ferroptosis and the metabolic state of the tumor microenvironment. In non-small cell lung carcinoma, HNRNPA2B1 promotes lactate accumulation by stabilizing lactate dehydrogenase A (LDHA) mRNA, which not only enhances resistance to ferroptosis but also directly inhibits the secretion of interferon-gamma (IFN-γ) by CD8^+^ T cells (Zhang Y. et al., 2025) (Figure 4A). Meanwhile, neutrophil extracellular traps (NETs) induce YTHDF2 to degrade SLC2A3 mRNA, thereby weakening CD8^+^ T cell function and blocking ferroptosis (Xu et al., 2025). Research targeting METTL5 revealed two independent pathways: in ovarian cancer, METTL5 promotes ATF4 translation and upregulates SLC7A11/SLC3A2 through unique 18S rRNA modifications, directly resisting T cell-induced ferroptosis (Hou et al., 2025) (Figure 4B). In GC, METTL5 relies on IGF2BP1 to stabilize NRF2 mRNA, reducing the tumor’s sensitivity to killing by CD8^+^ T cells through antioxidant mechanisms (Li X. et al., 2024) (Figure 4C). In summary, intervening in the aforementioned different m6A regulatory factors and their downstream metabolic or antioxidant targets is expected to restore tumor sensitivity to ferroptosis and reshape the immune surveillance function of CD8^+^ T cells.

**FIGURE 4:**
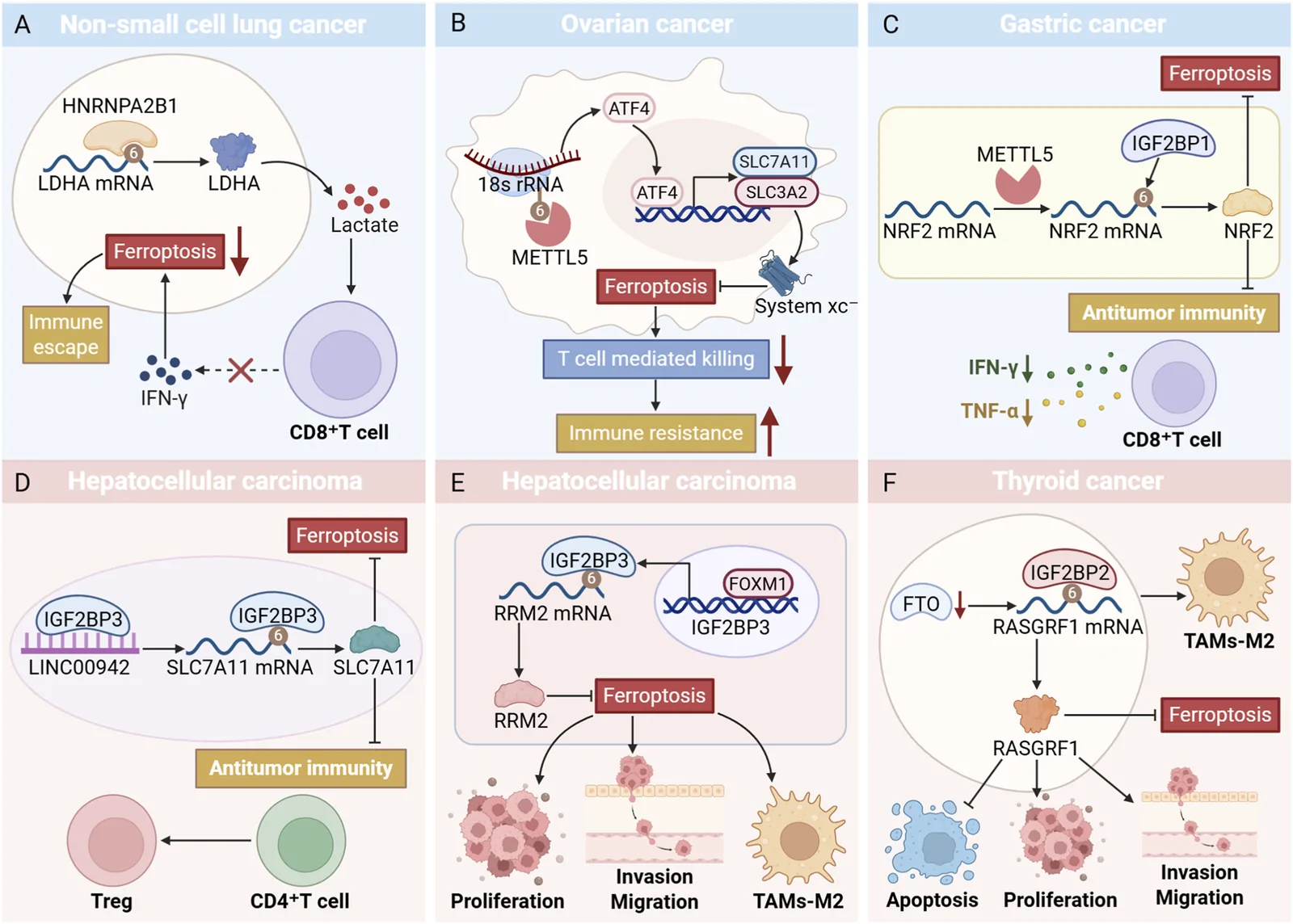
M6A regulates ferroptosis to reshape the tumor immune microenvironment. The m6A modification dynamically affects the TIME by regulating key nodes of ferroptosis. **(A–C)** At the CD8^+^ T cell level, HNRNPA2B1 promotes lactate accumulation by stabilizing LDHA mRNA, directly inhibiting CD8^+^ T cell function and enhancing tumor resistance to ferroptosis. NETs weaken T cell activity by degrading SLC2A3 mRNA through YTHDF2. METTL5 upregulates SLC7A11 or NRF2 through different mechanisms, reducing tumor sensitivity to T cell killing. **(D)** At the regulatory T cell level, the LINC00942/IGF2BP3 axis inhibits ferroptosis by stabilizing SLC7A11 mRNA while promoting Treg cell differentiation, exacerbating immunosuppression. **(E,F)** At the macrophage level, the FOXM1/IGF2BP3 axis inhibits ferroptosis and drives M2 polarization by stabilizing RRM2, while FTO inhibits M2 polarization by reducing RASGRF1 stability.

#### 4.2.2 Treg cells

Treg cells play an immunosuppressive role in the tumor microenvironment, and their function is directly regulated by the m6A modification, which indirectly affects the immunosuppressive phenotype through the metabolic pathway of ferroptosis. In HCC, the LINC00942/IGF2BP3 axis stabilizes SLC7A11 mRNA, promoting the differentiation of inducible Treg cells while inhibiting ferroptosis, thereby exacerbating tumor immunosuppression (Jin et al., 2024) (Figure 4D). Targeting the LINC00942/IGF2BP3/SLC7A11 axis can restore ferroptosis and reverse immunosuppression, providing a new direction for the treatment of liver cancer.

#### 4.2.3 Macrophages

Macrophages as innate immune phagocytes are precisely regulated by the m6A modification in their polarization and function within the tumor microenvironment. They participate in tumor progression by affecting the sensitivity of tumor cells to ferroptosis. In HCC, the FOXM1/IGF2BP3 axis drives tumor-associated macrophages (TAMs) polarization towards the M2 phenotype by stabilizing ribonucleotide reductase regulatory subunit M2 (RRM2) mRNA and inhibiting ferroptosis (Gao et al., 2025) (Figure 4E), whereas in thyroid cancer, FTO exerts an inhibitory effect on macrophage M2 polarization by reducing the stability of Ras protein-specific guanine nucleotide-releasing factor 1 (RASGRF1) mRNA (Li Z. et al., 2025) (Figure 4F). These studies collectively indicate that targeting m6A-related regulatory axes could provide potential therapeutic strategies for reshaping the TIME by intervening in the macrophage polarization and ferroptosis processes.

As shown in Figure 4, m6A inhibits CD8^+^ T cell function and enhances their resistance to ferroptosis by regulating lactate metabolism and System Xc^−^ activity. Additionally, it promotes Treg differentiation by inhibiting ferroptosis and modulates macrophage M2 polarization. Notably, the aforementioned m6A-ferroptosis regulatory network does not operate in isolation on individual immune cell subsets, but rather establishes interconnected immunosuppressive loops through multiple mechanisms. In HCC, for instance, the LINC00942/IGF2BP3 axis promotes Treg differentiation and suppresses CD8^+^ T cell infiltration while inhibiting ferroptosis in tumor cells, thereby establishing a positive “ferroptosis resistance-immunosuppression” feedback loop (Jin et al., 2024). Concurrently, M2 macrophage polarization, via the secretion of cytokines such as TGF-β and IL-10, synergistically facilitates Treg differentiation and suppresses CD8^+^ T cell activation (Zhang M. et al., 2026). This multicellular synergistic interplay suggests that targeting core nodes of the m6A-ferroptosis axis (such as SLC7A11/GPX4 or key m6A regulators) may simultaneously reverse multiple immunosuppressive mechanisms and drive systematic TIME remodeling.

Beyond local immune cells in the tumor microenvironment, recent studies have begun to uncover a tripartite regulatory mechanism involving the gut microbiota, host m6A modification, and cell-specific ferroptosis. A two-sample Mendelian randomization study based on single-cell eQTL data from 14 immune cell types demonstrated that host m6A modification modulates the composition of the gut and skin microbiota, thereby driving chronic disease progression through cell-specific ferroptosis (Zhang J. et al., 2026). Given the important role of the gut microbiota in modulating immunotherapy responses and the pivotal role of the m6A-ferroptosis axis in tumor immune evasion, future research should focus on whether microbial metabolites (such as short-chain fatty acids, bile acids, and tryptophan derivatives) regulate m6A modification patterns in tumor-infiltrating immune cells, thereby offering new avenues for cancer research.

### 4.3 Mediated treatment resistance

Tumors often exhibit treatment resistance during chemotherapy, radiation therapy, and targeted therapy, which is a major challenge in clinical tumor management. According to recent studies, m6A plays a key role in tumor treatment resistance by regulating the expression of ferroptosis-related genes.

#### 4.3.1 Chemotherapy resistance

Chemotherapy resistance is one of the core clinical challenges faced in current tumor therapy. Traditional drug resistance mechanisms often focus on the overexpression of drug efflux pumps, tumor heterogeneity, epigenetic changes, interactions of signaling pathways, and the tumor microenvironment (Pastan and Gottesman, 1991; Ge M. et al., 2025). However, tumor cells have been found to gain a survival advantage by evading ferroptosis, which has become a new field of interest in the formation of drug resistance.

Although the efficacy of cisplatin partly stems from inducing ferroptosis in tumor cells (Zhang et al., 2020), these cells can dynamically reprogram their m6A modification network to precisely regulate key nodes of the ferroptosis pathway, thereby weakening drug sensitivity and acquiring resistance. Specifically, in GC, the METTL3/YTHDC1 axis stabilizes SLC7A11 by overexpressing family with sequence similarity 120A (FAM120A) (Niu et al., 2024); in muscle-invasive bladder cancer (MIBC), the hypoxic environment stabilizes insulin-like growth factor-2 mRNA-binding protein 2 (IMP2/IGF2BP2) through LINC00941, which in turn synergistically upregulates importin 4 (IPO4) and SLC7A11 (Yan et al., 2024); in laryngeal cancer, RBM15, via IGF2BP3-dependent m6A modification, regulates the lysine (K)-specific demethylase 5B (KDM5B)/Fer-1 like family member 4 (FER1L4)/KCNQ1 overlapping the transcript 1 (KCNQ1OT1) axis to modulate the expression of GPX4/ACSL4 (Liang et al., 2024). These changes collectively enhance the ability of cells to clear lipid peroxides, directly inhibiting ferroptosis and ultimately leading to resistance to cisplatin chemotherapy. Some resistance mechanisms also involve metabolic reprogramming. Exosomes carrying miR-130a-3p and miR-4443 can inhibit METTL14 or METTL3 via targeting, interfering with the m6A modification-dependent regulation of FSP1 expression, thereby inhibiting ferroptosis and inducing cisplatin resistance (Han H. et al., 2025; Song et al., 2021). Furthermore, in LUAD, the METTL3-mediated degradation of FENDRR blocks TFRC-dependent iron uptake, thus limiting the Fenton reaction (Zhao et al., 2024). These mechanisms jointly form a multidimensional compensatory network that systematically enhances the ability of tumor cells to suppress ferroptosis, ultimately leading to resistance to cisplatin chemotherapy.

Similarly, the above mechanism is also widely present in the resistance to other chemotherapy drugs. Triple-negative BC relies heavily on chemotherapy due to the lack of specific therapeutic targets, but acquired resistance often leads to treatment failure (Serrano García et al., 2024; Egeland et al., 2024). In this context, targeting the m6A-ferroptosis axis provides a new strategy to break this clinical dilemma. Existing studies revealed that the ZC3H13/YTHDF2 axis can degrade lncRNA KCNQ1OT1 in an m6A-dependent manner, thereby relieving its transcriptional repression on TraB domain containing (TRABD), ultimately inducing ferroptosis and effectively reversing doxorubicin resistance (Huang et al., 2025). For pancreatic cancer, which is extremely difficult to cure due to high levels of fibrosis, gemcitabine resistance is influenced by multiple modification signals: on the one hand, USP10 stabilizes IGF2BP3 through deubiquitination and upregulates STEAP3 (Liang et al., 2026); on the other hand, O-GlcNAc glycosylation modification can stabilize the METTL3 protein, activating its function to degrade high mobility group box 1 (HMGB1) mRNA in a YTHDF2-dependent manner (Wang Q. et al., 2025). Both pathways maintain the drug-resistant phenotype of tumors by inhibiting ferroptosis. In GC, HNRNPC reshapes cell metabolism by upregulating monocarboxylate transporter 1 (MCT1) expression, utilizing enhanced aerobic glycolysis and lactic acid accumulation to construct a metabolic barrier, thereby resisting ferroptosis and leading to oxaliplatin resistance (Yang G. et al., 2025). These findings indicate that cisplatin resistance primarily involves the direct regulation of core anti-ferroptotic regulators such as SLC7A11 and GPX4, whereas resistance to doxorubicin and gemcitabine relies more on m6A reader-mediated post-transcriptional regulation (such as YTHDF2 and IGF2BP3) and metabolic remodeling, suggesting that reversal strategies should be tailored to both the drug type and tumor-specific regulatory networks.

#### 4.3.2 Radiation therapy resistance

Radiation therapy is the cornerstone of clinical treatment for various solid tumors, but the inherent or acquired radiation resistance of tumor cells often leads to local control failure and recurrence, which is a severe bottleneck limiting treatment efficacy (Shen R. et al., 2025). Increasing evidence suggests that the m6A modification of ferroptosis epigenetic regulation is a key variable determining the sensitivity of radiation therapy.

Radiation therapy is the curative method for NPC, whereas 10%–17% of patients still experience recurrence or metastasis due to radioresistance, which is a major cause of treatment failure (Miao et al., 2020). Recent studies have found that FTO maintains the stability of Otubain-1 (OTUB1) mRNA and upregulates its expression by erasing m6A modifications, thereby blocking ferroptosis signaling (Huang WM. et al., 2023), while METTL3 enhances the stability of SLC7A11 mRNA, promoting its protein expression (Dai et al., 2025). Both pathways enhance the antioxidant defense capability of tumor cells and inhibit radiation-induced ferroptosis, leading to radioresistance. Moreover, in HCC, METTL3 stabilizes SLC7A11 mRNA through the m6A/IGF2BP2 axis and concurrently suppresses its protein degradation via the m6A/YTHDF2/suppressor of the cytokine signaling 2 (SOCS2) axis, thereby synergistically maintaining antioxidant defense function and promoting radioresistance (Zhang C. et al., 2025). Similarly, in anaplastic thyroid carcinoma (ATC), YTHDF2 helps tumor cells evade ferroptosis by stabilizing the sterol regulatory element binding transcription factor 1 (SREBF1) and driving lipid remodeling (Dai et al., 2026). In NSCLC, HNRNPA2B1 regulates the hepatoma-derived growth factor (HDGF)/pleiotrophin (PTN) signaling pathway, mediating autophagy-dependent ferroptosis escape (Han F. et al., 2025). Targeting this pathway can inhibit autophagy and promote ferroptosis, providing a novel strategy to enhance the sensitivity of lung cancer to radiation therapy. Although specific regulators and downstream targets vary, these aforementioned mechanisms drive radioresistance by suppressing radiation-induced ferroptosis, suggesting that targeting key nodes of the m6A-ferroptosis axis (such as SLC7A11 and YTHDF2) may represent a common strategy to enhance radiosensitivity.

The m6A-ferroptosis axis is a key target for regulating tumor radiotherapy response, while its clinical translation faces the challenges of complex mechanisms and tumor type dependence. Future research needs to deeply analyze its dynamic network in the radiotherapy microenvironment and develop precise combination intervention strategies.

#### 4.3.3 Targeted therapy resistance

In tumor-targeted therapy resistance, the m6A modification also plays a key role in regulating ferroptosis. The resistance mechanism of human epidermal growth factor receptor 2 (HER2)-positive BC is complex, often involving HER2 receptor mutations, compensatory activation of bypass signaling pathways, and evasion of cell death (Hurvitz et al., 2013; Wang et al., 2022). Recent studies have found that the downregulation of METTL14 upregulates SLC7A11 and FPN1 by activating the fibroblast growth factor receptor 4 (FGFR4)/wingless/integrated (Wnt)/β-catenin axis, inhibiting ferroptosis and leading to resistance to anti-HER2 therapy (Zou et al., 2022).

The resistance mechanism of HCC to targeted drugs often involves the activation of bypass compensatory signaling, epigenetic regulation, and remodeling of the tumor microenvironment (Chen et al., 2024; Qin et al., 2024). Research has found that IGF2BP3 inhibits ferroptosis by recognizing the m6A modification of NRF2 mRNA and enhancing its stability, leading to the resistance of HCC to sorafenib (Lu et al., 2022). On the other hand, peroxisome proliferator-activated receptor-γ coactivator-1α (PPARGC1A) inhibits the WNT/β-catenin/bone morphogenetic protein and activin membrane-bound inhibitor (BAMBI) signaling axis, thereby regulating the transforming growth factor-beta (TGF-β)/small mothers against decapentaplegic (SMAD)/ACSL5 pathway, inhibiting ROS generation and promoting ferroptosis, thus enhancing the sensitivity of HCC to lenvatinib. This pathway is inhibited by METTL3/WTAP-m6A-YTHDF2 epigenetic modification, while metformin can restore PPARGC1A expression by inhibiting METTL3, providing a new combination strategy for HCC treatment (Zhang Q. et al., 2023).

In the targeted therapy of LUAD by epidermal growth factor receptor (EGFR)-tyrosine kinase inhibitors (TKIs), the m6A modification-mediated inhibition of ferroptosis is an important factor leading to resistance. Specifically, FTO effectively inhibits ferroptosis via blocking the miR-138-5p/lipocalin 2 (Lcn2) axis (Ding et al., 2024), while METTL16 directly upregulates GPX4 (Zeng et al., 2025), both inducing resistance of tumors to gefitinib and AZD-9291, suggesting that intervening in the m6A-ferroptosis pathway is a potential strategy to overcome multi-generation EGFR-TKIs resistance.

The above mechanisms indicate that targeting the key nodes of m6A regulation can restore sensitivity to ferroptosis, providing potential combination therapy strategies to reverse drug resistance in targeted therapy for various tumors. However, given that the m6A modification has a high degree of cell dependency and is indispensable in normal physiological processes, future research must further clarify the specificity of its regulatory network and overcome the potential off-target effects and systemic toxicity of small molecule drugs, in order to truly translate this “epigenetic-metabolic” linkage mechanism into safe and effective clinical applications.

In addition to the known regulatory mechanisms of ferroptosis associated with the aforementioned chemotherapy, radiotherapy, and targeted therapy modalities, as summarized in Table 1, recent studies have also revealed a previously undiscovered mutual regulatory relationship between ferroptosis and cuproptosis. In HCC, these two metal-driven death pathways share multiple regulatory nodes, including glutathione metabolism, mitochondrial function, and the interactive regulation of the NRF2/p53 axis. Notably, RNA m6A modification also serves as a shared epigenetic regulatory mechanism for both pathways (Wang L. et al., 2025). These findings suggest that m6A may serve as a common upstream regulator of multiple metal-dependent cell death pathways, a concept that has not been systematically explored in previous research. From a therapeutic standpoint, the concurrent activation of ferroptosis and cuproptosis is anticipated to yield synergistic anticancer effects, offering a promising strategy particularly for tumors resistant to single cell death pathways.

**TABLE 1:** The role of m6A-regulated ferroptosis in tumor therapy resistance.

Types	Regulators	Targets	Regulatory pathway	Clinical application	Tumor types	References
Writer	METTL3	SLC7A11	METTL3-m6A-YTHDC1-FAM120A-SLC7A11 pathway	Cisplatin resistance	Gastric cancer	Niu et al. (2024)
Reader	IMP2	SLC7A11	IMP2-IPO4/SLC7A11 pathway	Cisplatin resistance	Muscle-invasive bladder cancer	Yan et al. (2024)
Writer	RBM15	GPX4/ACSL4	KDM5B/FER1L4/KCNQ1OT1 axis	Cisplatin resistance	Laryngeal carcinoma	Liang et al. (2024)
Writer	METTL14	FSP1	miR-130a-3p/METTL14 axis	Cisplatin resistance	Esophageal cancer	Han et al. (2025a)
Writer	METTL3	FSP1	miR-4443/METTL3/FSP1 pathway	Cisplatin resistance	Non-small cell lung carcinoma	Song et al. (2021)
Writer	METTL3	TFRC	FENDRR/TFRC axis	Cisplatin resistance	Lung adenocarcinoma	Zhao et al. (2024)
Writer	ZC3H13	KCNQ1OT1	KCNQ1OT1/TRABD axis	Doxorubicin resistance	Triple negative breast cancer	Huang et al. (2025)
Reader	IGF2BP3	STEAP3	USP10-IGF2BP3-STEAP3 axis	Gemcitabine resistance	Pancreatic adenocarcinoma	Liang et al. (2026)
Writer	METTL3	HMGB1	OGT-METTL3-HMGB1 axis	Gemcitabine resistance	Pancreatic cancer	Wang et al. (2025c)
Reader	HNRNPC	MCT1	HNRNPC/MCT1 axis	Oxaliplatin resistance	Gastric cancer	Yang et al. (2025a)
Eraser	FTO	OTUB1	FTO/OTUB1 axis	Radioresistance	Nasopharyngeal carcinoma	Huang et al. (2023a)
Writer	METTL3	SLC7A11	METTL3/IGF2BP2/SLC7A11 axis	Radioresistance	Nasopharyngeal carcinoma	Dai et al. (2025)
Writer	METTL3	SLC7A11	m6A/YTHDF2/SOCS2 axis	Radioresistance	Hepatocellular carcinoma	Zhang et al. (2025c)
Reader	YTHDF2	SREBF1	YTHDF2-SREBF1 pathway	Radioresistance	Anaplastic thyroid carcinoma	Dai et al. (2026)
Reader	HNRNPA2B1	PTN	HNRNPA2B1/HDGF/PTN axis	Radioresistance	Non-small cell lung carcinoma	Han et al. (2025b)
Writer	METTL14	FGFR4	β-catenin/TCF4-SLC7A11/FPN1 axis	Anti-HER2 therapy	Breast cancer	Zou et al. (2022)
Reader	IGF2BP3	NRF2	IGF2BP3-NRF2 axis	Sorafenib resistance	Hepatocellular carcinoma	Lu et al. (2022)
Writer	METTL3/WTAP	ACSL5	PPARGC1A/BAMBI/ACSL5 axis	Lenvatinib resistance	Hepatocellular carcinoma	Zhang et al. (2023b)
Eraser	FTO	LCN2	miR-138-5p/LCN2 axis	Gefitinib resistance	Lung adenocarcinoma	Ding et al. (2024)
Writer	METTL16	GPX4	METTL16/GPX4/Ferroptosis axis	AZD-9291 resistance	Lung adenocarcinoma	Zeng et al. (2025)

## 5 Clinical transformation of the m6A regulation of tumor ferroptosis

The interaction between m6A modification and ferroptosis constitutes a key part of tumor metabolic reprogramming, providing a new perspective for breaking through existing treatment bottlenecks. In-depth explorations of this regulatory network not only help in accurately assessing patients’ survival prognosis and drug susceptibility but also provides a theoretical basis for developing new anti-tumor strategies. On this basis, this section focuses on clinical translation, providing a systematic discussion centered on three major translational tiers: the discovery of prognostic biomarkers and clinical risk stratification; the development of preclinical therapeutic agents, including natural compounds and synthetic small molecules; and the translational challenges encountered in clinical trials.

### 5.1 Prognostic biomarkers

The exploration of biomarkers based on the interaction between m6A modification and ferroptosis provides a new direction for tumor prognosis assessment and precise stratification. Multiple studies have utilized multi-omics integration and machine learning algorithms to screen key m6A-ferroptosis-related genes or lncRNAs and construct multifactorial prognostic models for various malignancies such as cervical neoplasms and LUAD. These models not only demonstrate excellent survival prediction efficacy but also reveal a close association between high-risk scores and immune-suppressive microenvironments, DNA replication activity, and drug sensitivity (such as responses to immunotherapy or chemotherapy) (Liu J. et al., 2025; Wen et al., 2025; Li et al., 2023; He et al., 2025; Wu et al., 2022). Furthermore, independent validation studies further confirmed that core molecules such as IGF2BP3 in LUAD (Xu X. et al., 2022), ZFP69B in HCC (He et al., 2025), and LDHA in EC (Huang et al., 2024) can drive tumor progression by stabilizing the expression of anti-ferroptosis genes or reshaping the metabolic-immune network, serving as independent biomarkers with significant clinical implications.

Multi-omics modeling based on large-scale public databases has further enhanced the potential for clinical translation of the above findings. In LUAD, Gao et al. developed a prognostic model comprising 19 ferroptosis-related lncRNAs based on 504 The Cancer Genome Atlas (TCGA) samples, achieving a 1-year area under the curve (AUC) of 0.763 in internal validation, and revealed expression differences in 34 immune checkpoint genes and 13 m6A-related genes between high-risk and low-risk groups (Gao C. et al., 2022). In GC, Ma et al. integrated m6A modification and ferroptosis gene sets to construct a six-lncRNA prognostic model, demonstrating that the 3-year survival rate in the high-risk group was only 27.8% (compared to 54.8% in the low-risk group), and that the high-risk group exhibited an immunosuppressive microenvironment characterized by reduced CD8^+^ T cell infiltration and an enrichment of M2 macrophages and Tregs (Ma et al., 2026). These multi-omics models based on large-scale cohorts provide important support for the clinical translation of m6A-ferroptosis-related biomarkers.

In summary, these findings not only enrich our understanding of the molecular mechanisms of tumor progression but also provide an important theoretical basis and potential targets for risk stratification management and individualized treatment strategies for patients. Although these biomarkers demonstrate high predictive performance in computational biology, their clinical translation faces significant obstacles: most existing models are derived from retrospective public databases and lack validation in prospective, multi-center, large-scale clinical cohorts.

### 5.2 Treatment targets

Given the precise regulation of ferroptosis by m6A, targeting the m6A-ferroptosis interaction network has become a new strategy for tumor treatment. Drug intervention on m6A regulatory factors or upstream signals can disrupt redox homeostasis and induce lipid peroxidation. This section focuses on discussing two types of drugs: natural compounds and synthetic small molecules, exploring how they trigger ferroptosis by reshaping the m6A modification, providing a basis for the development of new anti-cancer drugs and overcoming drug resistance.

#### 5.2.1 Natural compounds

Curdione is a diterpenoid compound extracted from Curcumae Rhizoma, exhibiting anti-inflammatory and anti-tumor activities (Yang X. et al., 2025). Studies have established that in CRC, curdione enhances the m6A modification of SLC7A11/SLC3A2 and homeobox A13 (HOXA13) via upregulating METTL14 and YTHDF2, promoting their degradation and thereby inducing ferroptosis and inhibiting tumor growth (Wang F. et al., 2023). Gambogic acid (GA) is another class of natural compounds with anti-tumor effects (Wang et al., 2009; Yang et al., 2007), which downregulates METTL3 in CRC, reducing the m6A modification level of p62 mRNA, thereby inhibiting the stabilization of p62 mRNA by IGF2BP1, ultimately inducing ferroptosis and inhibiting tumor progression (Wang et al., 2025d).

Platycodin D (PD) is an active ingredient extracted from *Platycodon grandiflorum*, with various functions including antioxidant, anti-tumor and immune regulation (Yoon et al., 2026; Yang et al., 2026). In prostate cancer, PD induces ferroptosis and enhances tumor cell sensitivity to docetaxel (DTX) by downregulating METTL16, which modifies nuclear protein 1 (NUPR1) mRNA with m6A (Sun et al., 2025).

Mupirocin is a natural antibiotic isolated from the fermentation product of *Pseudomonas fluorescens* (Thomas et al., 2010). Recent studies have found that it can act as a novel FTO inhibitor, downregulating the expression of SLC7A11 and GPX4 through an m6A-YTHDF2 dependent mechanism, thereby restoring the sensitivity of CRC cells to ferroptosis and effectively inhibiting tumor progression (Qiao et al., 2024).

Licochalcone A (Lico A) is a natural flavonoid compound with antioxidant and anti-tumor activities (Liu et al., 2025d). In acute myeloid leukemia (AML), Lico A can activate the p53 pathway by downregulating IGF2BP3, inhibiting its stabilizing effect on murine double minute 2 (MDM2) mRNA, ultimately inducing ferroptosis and inhibiting tumor cell proliferation (Han et al., 2024).

Astragaloside IV, isolated from the traditional Chinese medicine *Astragali Radix*, has been shown to directly bind to FTO and regulate FTO-mediated m6A methylation, thereby inducing ferroptosis in GC (Yin et al., 2026). Artesunate, derived from *Artemisia annua*, enhances WTAP-mediated m6A modification, promotes YTHDC2-dependent ATG5 translation, and triggers autophagy-dependent ferroptosis, demonstrating therapeutic potential in HCC (Li Y. et al., 2026). Furthermore, Curculigoside has been reported to induce ferroptosis in NSCLC by regulating WTAP-mediated GCH1 m6A modification (Lv et al., 2026).

These studies indicate that natural active ingredients from different sources can regulate the ferroptosis pathway by targeting m6A regulatory factors, providing a basis for the development of anti-cancer drugs based on the m6A-ferroptosis axis.

#### 5.2.2 Synthesis of small-molecule regulators

Inhibitors targeting m6A regulatory factors have been confirmed to exert anti-tumor activity by intervening in the ferroptosis pathway. In gliomas, the synthetic small molecule EF24 inhibits METTL3, disrupting the m6A-YTHDF1-NRF2 translation enhancement complex formed with YTHDF1, thereby downregulating the expression of NRF2 and GPX4, ultimately inducing ferroptosis and inhibiting tumor growth (Yang Y. et al., 2025). Furthermore, in radioresistant NPC, the high expression of FTO enhances the stability of OTUB1 by mediating m6A demethylation, thereby weakening radiotherapy-induced ferroptosis, while the FTO inhibitor FB23-2 can block this mechanism and restore tumor sensitivity to radiotherapy (Huang WM. et al., 2023). Notably, the abnormal regulation of m6A modification is also involved in chemotherapy-related toxicity processes. Doxorubicin activates c-Jun to upregulate METTL3 in cardiomyocytes, thereby stabilizing TFRC mRNA in an m6A-IGF2BP2-dependent manner, leading to iron accumulation and ferroptosis, and causing cardiac toxic side effects. Meanwhile, the highly selective METTL3 inhibitor STM2457 can reverse this process, providing a potential therapeutic direction for the prevention and treatment of doxorubicin-induced cardiac toxicity (Wu et al., 2024). These studies collectively indicate that inhibitors targeting m6A regulatory factors can play an important role in tumor treatment and toxicity management by precisely intervening in the m6A modification of ferroptosis-related genes.

In addition to directly targeting m6A regulatory factors, small molecule synthesis can also regulate ferroptosis in an m6A modification-dependent manner by intervening in upstream signaling pathways. For example, PMX205 blocks the extracellular signal-regulated kinase 1/2 (ERK1/2) pathway by targeting complement 5a receptor 1 (C5aR1), downregulating METTL3-mediated GPX4 mRNA m6A modification and stability, thereby inducing ferroptosis in glioblastoma (Meng et al., 2024). In triple-negative BC, to address the ferroptosis resistance caused by the 26S proteasome non-ATPase regulatory subunit 14 (PSMD14)-driven splicing factor 3b subunit 4 (SF3B4)/HNRNPC complex upregulating fatty acid desaturase 1 (FADS1) and the Akt/mammalian target of rapamycin (mTOR) pathway, the combined use of PSMD14 inhibitor (O-phenanthroline) and exogenous arachidonic acid can exert a synergistic anti-tumor effect, providing a novel strategy for the treatment of related tumors (Yu et al., 2025).

Classic ferroptosis inducers have also been confirmed to exert a synergistic anti-tumor effect via the “upstream signal-m6A-ferroptosis” axis. In gliomas, erastin downregulates lncRNA SNAI3-AS1, relieving its competitive binding to staphylococcal nuclease and tudor domain containing 1 (SND1), thereby weakening the ability of SND1 to stabilize Nrf2 mRNA in an m6A-dependent manner, ultimately promoting ferroptosis (Zheng et al., 2023). In addition, erastin can upregulate Yin Yang 1 (YY1), promote METTL3 expression and subsequently regulate the transcription of SLC7A11 through GATA binding protein 3 (GATA3) and KDM6B, synergistically inducing ferroptosis and inhibiting tumor progression in lung cancer, cervical neoplasms and BC (Zhang et al., 2025d).

In summary, targeting m6A and its upstream signaling pathways to regulate ferroptosis provides a new strategy for improving the efficacy of cancer treatment. Table 2 summarizes representative natural compounds and synthetic small-molecule drugs targeting m6A regulatory factors. However, given the widespread nature of the m6A modification, determining how to achieve precise delivery to tumor tissues while avoiding interference with normal cells remains a significant challenge for future applications.

**TABLE 2:** Molecules targeting m6A regulators and their clinical applications.

Compound	Targets	Expression	Inhibits/promotes ferroptosis	Biological behavior changes	Tumor types	References
Curdione	METTL14/YTHDF2	Upregulation	Promote	Inhibits tumor growth	Colorectal cancer	Wang et al. (2023b)
Gambogic aci (GA)	METTL3	Downregulation	Promote	Inhibits tumor growth	Colorectal cancer	Wang et al. (2025d)
Platycodin D (PD)	METTL16	Downregulation	Promote	Enhances tumor cell sensitivity to docetaxel	Prostate cancer	Sun et al. (2025)
Mupirocin	FTO	Downregulation	Promote	Inhibits tumor growth	Colorectal cancer	Qiao et al. (2024)
Licochalcone A (Lico A)	IGF2BP3	Downregulation	Promote	Inhibits tumor cell proliferation	Acute myeloid leukemia	Han et al. (2024)
EF24	METTL3	Downregulation	Promote	Inhibits tumor growth	Glioma	Yang et al. (2025c)
FB23-2	FTO	Downregulation	Promote	Overcomes tumor radioresistance	Nasopharyngeal carcinoma	Huang et al. (2023a)
STM2457	METTL3	Downregulation	Inhibit	Prevents doxorubicin-induced cardiotoxicity	—	Wu et al. (2024)

#### 5.2.3 Comparison of different intervention strategies

Natural compounds possess advantages in structural diversity, multi-targeting capacity, and biosafety, with most originating from traditional medicine-food homology practices. However, their clinical translation is significantly hindered by poor water solubility, low oral bioavailability, insufficient target specificity, and batch-to-batch compositional variation (Kaur et al., 2026). In contrast, synthetic small-molecule inhibitors exhibit superior target selectivity, low-nanomolar binding potency, and well-defined structure-activity relationships, yet face challenges including off-target toxicity, narrow therapeutic windows, and secondary resistance mutations induced by long-term administration (Jaguan et al., 2024). Currently, the vast majority of evidence remains at the preclinical stage, with only very few candidates (such as the METTL3 inhibitor STM2457) having entered early exploratory trials.

Beyond small molecule regulators, emerging nanocatalytic materials offer a complementary strategy for targeting the m6A-ferroptosis axis. Single-atom nanozymes (SANs), by virtue of their atomically dispersed metal active sites and high-efficiency catalytic performance, can dynamically regulate ROS cascades and precisely remodel the redox microenvironment and macrophage polarization phenotypes in different pathological models (Zhu et al., 2026; Li J. et al., 2026). Cell membrane biomimetic coating strategies further enhance the targeting and biocompatibility of nanosystems, enabling precise modulation of programmed cell death pathways such as ferroptosis and pyroptosis (Lin et al., 2026). Furthermore, microenvironment-responsive intelligent nanoplatforms have been demonstrated to effectively reverse the immunosuppressive TIME and sensitize tumor immunotherapy by remodeling macrophage phenotypes and mobilizing CD8^+^ T cell infiltration (Huang R. et al., 2023).

Notably, a potential synergistic effect exists between the aforementioned nanomaterial-mediated exogenous ferroptosis induction strategy and the endogenous m6A epitranscriptomic regulatory mechanism: the expression levels of core anti-ferroptosis targets such as SLC7A11 and GPX4, which are regulated by m6A modification, directly determine the sensitivity threshold of tumor cells to nanozyme-induced oxidative stress and ferroptosis. Meanwhile, the oxidative stress triggered by nanozymes may also exert feedback regulation on the activity of m6A modification enzymes and downstream m6A modification patterns, but the precise regulatory network remains to be elucidated. Combining m6A-targeting molecules with microenvironment-responsive nanocatalytic and delivery platforms holds promise for achieving spatiotemporal synergy between “metabolic ferroptosis induction” and “activation of the anti-tumor immune microenvironment,” providing a cutting-edge translational paradigm for overcoming tumor drug resistance.

### 5.3 Challenges in clinical translation

Despite significant efficacy in preclinical studies, no therapy targeting the m6A-ferroptosis axis has yet successfully advanced to phase II/III clinical oncology trials. This translational gap is primarily attributed to three core challenges. First, the pleiotropic nature of m6A modification poses major safety concerns: systemic inhibition of m6A regulators may disrupt normal physiological processes, leading to off-target toxicity (Liu C. et al., 2022). Second, most m6A modulators and ferroptosis inducers exhibit rapid *in vivo* clearance, low tumor enrichment, and poor penetration of physiological barriers (Zhu et al., 2025). Promising strategies to overcome these delivery barriers include the integration of microenvironment-responsive nanodelivery systems with single-atom nanozymes, though these approaches remain largely experimental. Third, the lack of prospectively validated companion diagnostic tools hinders precise identification of patient populations likely to benefit and prediction of drug resistance or toxicity risks. Addressing these challenges necessitates the integration of single-cell multi-omics analysis, advanced delivery technologies, and biomarker-driven clinical trial design to fully harness the therapeutic potential of the m6A-ferroptosis axis.

## 6 Conclusion

This review systematically elucidates the dual role of the m6A-ferroptosis pathway in promoting and inhibiting tumor growth and its capacity to reshape the tumor immune microenvironment, thereby influencing the efficacy of chemotherapy, radiation therapy, and targeted therapy. Existing evidence indicates that m6A modification modulates susceptibility to ferroptosis through multi-level regulatory mechanisms: from mRNA stability and splicing, to translation processes and demethylation reactions, ultimately impacting the function of key antioxidant proteins such as SLC7A11 and GPX4. This regulatory mechanism not only enhances our understanding of tumor progression and drug resistance but also identifies potential therapeutic targets for precision cancer therapy.

Despite these advances, two unresolved core paradoxes in this field severely hinder clinical translation. First, m6A regulators exhibit tissue specificity and context dependence, with the same regulator potentially producing opposite effects in different tumor types, genetic backgrounds, or microenvironments, making the development of universal management strategies extremely difficult. Second, the m6A–ferroptosis regulation is bidirectional, capable of both promoting and suppressing tumors, and nonspecific global activation or inhibition may lead to unpredictable or even contradictory outcomes in different malignancies. Resolving these paradoxes requires abandoning a “one-size-fits-all” approach and shifting toward precise regulation based on specific contexts and biomarkers.

To address these challenges, future research should be conducted at four levels. First, at the technical level, there is a need to promote a paradigm shift from bulk analysis to single-cell and spatial resolution studies. Current methods based on bulk tissue analysis cannot capture the cellular heterogeneity and spatial architecture of the tumor microenvironment. Integrating single-cell sequencing with spatial transcriptomics is expected to reveal the co-localization features of m6A modification and lipid peroxidation at single-cell and spatial *in situ* levels. The m6A-isoSC-seq technology developed by Ren et al. has provided a technical paradigm for this (Ren Z. et al., 2025). Such high-resolution methods are crucial for deciphering the functional heterogeneity of m6A regulatory factors in cancer stem cells, cancer-associated fibroblasts, and immune effector cells. Second, at the mechanistic level, the focus should be on the spatiotemporal dynamics and adaptive remodeling of m6A–ferroptosis regulation. Both ferroptosis and m6A modification exhibit dynamic plasticity. Tumor cells can evade ferroptosis and acquire drug resistance by reprogramming the m6A modification landscape under stress conditions such as chemoradiotherapy or immunotherapy. Future studies should combine longitudinal clinical samples with dynamic models to map the evolutionary trajectory of this regulatory axis throughout the entire treatment cycle. Third, at the clinical translation level, three interrelated challenges need to be addressed: biomarker-guided patient stratification, systematic evaluation of combination therapy regimens, and optimization of drug developability. This includes identifying biomarkers with prognostic or predictive value to distinguish responders from at-risk populations, assessing the synergistic effects of m6A inhibitors with immunotherapy or ferroptosis inducers with conventional therapy, and addressing issues such as low bioavailability, insufficient target specificity, and off-target toxicity. Fourth, at the model optimization level, the gap between preclinical research and clinical application must be bridged. Current mouse models cannot fully recapitulate the heterogeneity and microenvironmental complexity of human tumors. Introducing patient-derived organoids (PDOs) and patient-derived xenograft (PDX) models (Vlachogiannis et al., 2018; Liu et al., 2023), together with longitudinal clinical sample collection, can facilitate the optimization of patient stratification and trial design, accelerating the clinical translation of m6A–ferroptosis-targeted therapies. The validation of the METTL16-SENP3-LTF pathway in HCC has demonstrated the feasibility of this approach (Wang J. et al., 2024).

In summary, the interplay between m6A and ferroptosis constitutes a promising yet challenging research avenue in the field of precision oncology. By systematically addressing issues arising from tissue-specific differences and bidirectional regulatory mechanisms, advancing technological capabilities, elucidating various dynamic mechanisms, prioritizing biomarker-based clinical application research, and optimizing preclinical models, this field is expected to develop new therapeutic strategies. These strategies could leverage the vulnerabilities of ferroptosis to target diverse malignancies, thereby opening new avenues for precision cancer therapy.

## Glossary

3′UTR: 3′untranslated regions
ALKBH5: AlkB homolog 5
ACSL4: Acyl-CoA synthetase long-chain family member 4
ACC: Acetyl-CoA carboxylase
ALOXs: Arachidonic acid lipoxygenases
AML: Acute myeloid leukemia
ATC: Anaplastic thyroid carcinoma
BC: Breast cancer
CRC: Colorectal cancer
DHODH: Dihydroorotate dehydrogenase
eIF3: Eukaryotic initiation factor 3
EC: Endometrial cancer
EGFR: Epidermal growth factor receptor
FTO: Fat mass and obesity-associated protein
FMRP: Fragile X Messenger Ribonucleoprotein 1
FSP1: Ferroptosis suppressor protein 1
GSH: Glutathione
GPX4: Glutathione peroxidase 4
GCLC: Glutamate-cysteine ligase catalytic subunit
GSS: Glutathione synthetase
GC: Gastric cancer
HMGB1: High mobility group box 1
HER2: Human epidermal growth factor receptor 2
HCC: Hepatocellular carcinoma
IGF2BPs: Insulin-like growth factor 2 mRNA-binding proteins
ICIs: Immune checkpoint inhibitors
LIP: Labile iron pool
LPCAT3: Lysophosphatidylcholine acyltransferase 3
LUAD: Lung adenocarcinoma
MVBs: Multivesicular bodies
MIBC: Muscle-invasive bladder cancer
MTC: Methyltransferase complex
METTL3: Methyltransferase-like 3
M6A: N6-methyladenosine
NCOA4: Nuclear receptor coactivator 4
NADPH: Nicotinamide adenine dinucleotide phosphate
NSCLC: Non-small cell lung cancer
Nrf2: Nuclear factor erythroid 2-related factor 2
NPC: Nasopharyngeal carcinoma
NETs: Neutrophil extracellular traps
PCD: Programmed cell death
PL-PUFAs: Phospholipid-polyunsaturated fatty acids
PKA: Protein kinase A
PDOs: Patient-derived organoids
PDX: Patient-derived xenograft
ROS: Reactive oxygen species
RBM15: RNA-binding motif protein 15
STEAP3: Six-transmembrane epithelial antigen of the prostate 3
SLC11A2: Solute carrier family 11 member 2
STAT3: Signal transducer and activator of transcription 3
TFR1: Transferrin receptor 1
TAMs: Tumor-associated macrophages
TKIs: Tyrosine kinase inhibitors
TIME: Tumor immune microenvironment
VIRMA: Vir-like m6A methyltransferase-associated
WTAP: Wilms tumor 1-associated protein
ZC3H13: Zinc finger CCCH domain-containing protein 13
